# Total Protein and 10‐Hydroxy‐2‐Decenoic Acid Content, In Vitro Bioaccessibility, Antibacterial, and Antidiabetic Properties of Royal Jelly Collected at Different Harvesting Times

**DOI:** 10.1002/fsn3.72127

**Published:** 2026-07-29

**Authors:** Eyuel Welelaw, Abera Belay, Tadele Alemu, Teferi Damto, Admasu Addi, Mesfin Getachew Tadesse, Endale Amare, Feriehiwote Weldeyohanis Gebremariam, Ibrahim Ahmed Sherif, Henock Woldemichael Woldemariam

**Affiliations:** ^1^ Department of Food Science and Post‐Harvest Technology Wachemo University, College of Agricultural Science Hossana Ethiopia; ^2^ Department of Food Science and Applied Nutrition Addis Ababa Science and Technology University Addis Ababa Ethiopia; ^3^ Biotechnology and Bioprocess Center of Excellence Addis Ababa Science and Technology University Addis Ababa Ethiopia; ^4^ Oromia Agricultural Research Institute, Holeta Bee Research Center Holeta Ethiopia; ^5^ Department of Industrial Chemistry Addis Ababa Science and Technology University Addis Ababa Ethiopia; ^6^ Nutrition, Environmental Health and Non‐Communicable Diseases Research Directorate Ethiopian Public Health Institute Addis Ababa Ethiopia; ^7^ Department of Chemical Engineering Addis Ababa Science and Technology University Addis Ababa Ethiopia

**Keywords:** antibacterial, antidiabetic, bioaccessibility, biological function, protein, royal jelly

## Abstract

Royal jelly has received interest as a functional food. Consumers are interested in functional foods, particularly from natural sources. Royal jelly's protein and 10‐hydroxy‐2‐decenoic acid content and biological activities are affected by a number of factors. This study aimed to evaluate the protein and 10‐hydroxy‐2‐decenoic acid content, in vitro antibacterial, antidiabetic properties, and bioaccessibility of royal jelly collected at 72 and 144 h after larval grafting; and were examined using standard methods*.* Royal jelly collected at the 72 h showed higher protein (12.99%) and 10‐hydroxy‐2‐decenoic acid (3.39%) content. The bioaccessibility of protein and 10‐hydroxy‐2‐decenoic acid content in royal jelly in the gastric stage was found to be lower compared to intestinal stage for both 72 and 144 h. The inhibitory concentration (IC_50_) was highest against *Bacillus cereus* (33.15%), while the lowest potency was observed in *Salmonella enterica* (50.10%) at 72 h. The high IC_50_ potency was found at 144 h in *Bacillus cereus* (49.09%), and the lowest was in *Listeria monocytogenes* (63.25%). The maximal inhibition of α‐amylase (75.33%) and α‐glucosidase (75.93%) was observed to have significant difference (*p* < 0.05) in royal jelly collected at 72 h. For royal jelly collected at 72 and 144 h, the IC_50_ values of α‐amylase were 24.70% and 33.87%, respectively, while the IC_50_ values of α‐glucosidase were 2.81% and 12.27%. Therefore, royal jelly collected at 72 h possesses high protein and is rich in 10‐hydroxy‐2‐decenoic acid content. It is also more bioaccessible, and exhibits strong antibacterial and antidiabetic effects, which are attributed to the variation in harvesting time.

## Graphical abstract

In this study, the total protein content and 10‐hydroxy‐2‐decenoic acid content were determined in royal jelly collected at the 72 and 144 h. In vitro bioaccessibility, antibacterial, and antidiabetic properties of royal jelly collected at the 72 and 144 h were also determined.

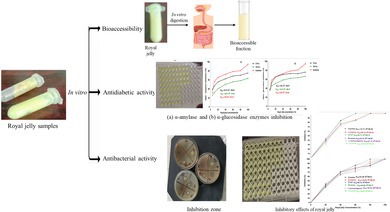

## 1 Introduction

Royal jelly is among the most significant bee products, which is secreted by worker honeybees' hypopharyngeal glands. It is known as a highly nutritious substance, containing major macronutrients, micronutrients and antioxidants (Maleki et al. 2019). Royal jelly has a distinct composition, unlike honey, pollen, or other bee products. More recently, sugar profiles, water content, protein and lipid contents tend to be accepted for the most common criteria used to characterize royal jelly quality (Bărnuţiu et al. 2011). Among these criteria, 10‐hydroxy‐2‐decenoic acid and total protein contents may also provide useful information on the quality of royal jelly. Multiple factors influence the 10‐hydroxy‐2‐decenoic acid and total protein contents of royal jelly, including seasonal and regional variability, botanical sources and bee species (Khalfan Saeed Alwali Alkindi et al. 2024).

Today, due to population growth and the limitations of modern medicine, there is a strong need for research into the introduction of new functional foods drived from natural origin is strongly needed to reduce antibiotic resistance and unwanted side effects of chemical agents (Mohammad et al. 2022). This issue is not only increasing the severity of diseases, but it is also increasing health care costs and mortality rates for certain diseases. To cope with this emerging challenge, research has made a significant effort to discover and develop new nutraceutical and functional foods that have better biological potential (Carpio et al. 2021).

In addition to providing nourishment, functional foods have been identified as a way to enhance human health by treating and preventing several diseases (Adefegha 2018). Natural ingredients have attracted attention for their broad‐spectrum antibacterial properties, especially those derived from honeybee products such as royal jelly (Turbatmath et al. 2025). Studies on royal jelly against a wide range of microorganisms worldwide show strong antimicrobial activity (Maželienė et al. 2022). According to Mohammad et al. (2022), bioactive compounds have an impact on the antibacterial properties of royal jelly samples that were collected from different geographical locations. Diabetes mellitus is a chronic metabolic disorder characterized by hyperglycemia and glucosuria either due to insufficient insulin secretion of the pancreas or due to insulin resistance of target cells (Jadon et al. 2024).

Determining a compound's bioaccessibility following digestion was the goal of numerous studies. The bioaccessibility of nutrients for absorption in the intestine is rather difficult and changes for a given food depending on processing conditions and interaction with other compounds, chemical status of the nutrient, release from the food matrix, suppressors or cofactors present in the food composition, formation of stable complexes that are gradually metabolized (Santos et al. 2019). The health advantages of natural functional foods are becoming more popular these days. Royal jelly's biological properties are related to its bioactive compounds, such as proteins, peptides, phenolic, and fatty acids (Bagameri et al. 2022). However, no investigation has been conducted on the impacts of harvesting time on the total proteins and 10‐hydroxy‐2‐decenoic acid content, bioaccessibility, antibacterial and antidiabetic activities of royal jelly, making the present study unique. Accordingly, this study examines royal jelly's total proteins and 10‐hydroxy‐2‐decenoic acid content, antibacterial, antidiabetic, and bioaccessibility at different harvesting times.

## 2 Materials and Methods

### 2.1 Study Design

To examine the total protein and 10‐hydroxy‐2‐decenoic acid content, in vitro antidiabetic, antibacterial properties and bioaccessibility of royal jelly, a complete randomized design (CRD) was employed. It had one factor (harvesting time of royal jelly) and two treatment levels (T1–72 h and T2–144 h) based on the modified method of Ma et al. (2021).

Yi=μ+HTi+εi

where *Yi* is the overall observations of the *i*th treatment, *μ* is the population means, *HTi* is the treatment effect of the *i*th harvesting time, and *εi* is the error term of the *i*th treatment and replication.

### 2.2 Sample Collection

Royal jelly was collected from the Holeta Bee Research Center, Ethiopia. It is located in Holeta town, 34 km away from Addis Ababa and is found at the geographical coordinates of Latitude 9.05723° or 9° 3′ 26″ north and Longitude 38.50442° or 38° 30′ 16″ east. Ten similar productive colonies of *Apis mellifera* honeybees were thus chosen at random and kept at the Holeta Bee Research Center. In order to ensure the best possible health and productivity, these experimental colonies were carefully maintained under closely controlled circumstances. Royal jelly was collected from *Apis mellifera* colonies using appropriate grafting materials from the queen cells. After a specific time, often between 72 and 144 h, the royal jelly‐filled cups are collected from the hive. Royal jelly samples for this investigation were taken at 72 and 144 h. For the royal jelly to be pure and of high quality, this procedure has been done precisely and stored at −80°C until analysis.

### 2.3 Determination of Total Protein Content

Total protein content of royal jelly was determined according to Bradford method using a UV–VIS Spectrophotometer (UV‐1900i) with minor modifications (El‐Guendouz et al. 2020; Popescu et al. 2009). Each royal jelly sample (250 mg) was suspended in 10 mL methanol/water (50/50; v/v) and sonicated for 60 min. Afterward, the pH was adjusted to 2.5 with phosphoric acid and the sample solutions were diluted 10 times. Standard protein is bovine serum albumin (BSA) used at a concentration of 1 mg/mL (100 μg/mL) in distilled water as a stock solution 1:1. A total of 5 mL of Bio‐Rad reagent diluted to 1:5 was added into 200 μL of royal jelly solutions and the mixture was well vortexed; then the absorbance was measured in a UV–VIS spectrophotometer (UV‐1900i) at 595 nm after 5 min incubation. The total protein content was expressed as percentage (%) using the bovine serum albumin standard curve (0.3–1.0 mg/mL) (Table S3).

### 2.4 Determination of 10‐Hydroxy‐2‐Decenoic Acid Content

The 10‐Hydroxy‐2‐decenoic acid (10‐HDA) was determined according to the procedure described by Ab Hamid et al. (2018) with slight modification. Briefly, 0.5 g of royal jelly sample was dissolved in 1 mL methanol. Then, 0.05 g anhydrous sodium sulphate was added into the solution to eliminate water before being centrifuged for 5 min at 4000 × g and adding the standard solutions in a concentration of 100 μg/mL. The samples were then analyzed using a Gas Chromatograph (Agilent‐7890 GC (G3442B))—MS spectrometer (Agilent 5977A MSD (G7037A)) with a DB‐5MS capillary column (30 × 0.25 mm, 0.25 μm film thickness). It was initially kept at 50°C for 2 min, rising at 20°C/min to 280°C, and holding for 10 min. Ionization energy was 70 eV and helium gas was applied to mediate the flow at a rate of 1.0 mL/min (Zhao et al. 2016). The volatile phytochemical compounds analysis was recognized based on mass spectral matching with National Institute Standard and Technology (NIST) libraries. Percentage peak area more than 0.08% was considered in this study. The results were presented as percentages (Table S2).

### 2.5 Determination of In Vitro Antibacterial Properties

The agar well diffusion method was used to examine each royal jelly sample's inhibitory effects on intestine pathogenic bacterial infections, following Humphries et al. (2021). By adding sterile distilled water, the total amount of the royal jelly increased to 2 mL and was properly mixed using a vortex. These royal jelly samples (50%, w/v) (50 mg/mL) were used to determine the antibacterial activity (Osés et al. 2016). Then, the antibacterial properties of royal jelly samples were tested against three pathogenic Gram‐positive bacteria ( *Bacillus cereus* NCCB100294, *Staphylococcus aureus* NCCB100294, *Listeria monocytogenes* NCCB100677), and three pathogenic Gram‐negative bacteria ( *Escherichia coli* NCCB72002, *Shigella sonnie* CCUg68726T, and *Salmonella enterica* NCCB100284). The bacteria used in this study were obtained from Bless Agri Foods Laboratory Service PLC. Bacterial samples from distinct colonies were transferred to Mueller Hinton Broth (MHB) medium following a 24 h incubation period. A positive control (bacteria without royal jelly) and a negative control (broth without bacteria) were included. Using the streak plate method, the selected pathogenic bacteria were seeded on Mueller Hinton Agar (MHA) media. At the end of the 18 h incubation period in the MHB medium, bacterial cultures were adjusted to 0.5 McFarland turbidity standards and used as an inoculum (Osés et al. 2016).

### 2.6 Screening for Antibacterial Activity

In vitro inhibitory activities of royal jelly samples were investigated by the agar well diffusion (AWD) method and the inhibitory activity of the samples was detected as a clear zone around the wells (Humphries et al. 2021). MHA was used in tests for antibacterial activity. After being cooled at room temperature, the autoclaved‐sterilized MHA mediums were promptly transferred in 25 mL volumes to sterile petri dishes. After the addition of the 500 μL inoculum, the petri dishes were cooled at room temperature for 1 h (Osés et al. 2016). After this period of time, a sterile cork borer was used to cut 5 mm diameter wells into the solidified mediums' surface. The 50 μL of the previously prepared royal jelly samples [50% (w/v)] was transferred to these wells and then the petri dishes were incubated at 37°C for 24 h.

A ruler was used to measure and record the observed inhibitory zones at the end of the incubation period. All tests were performed in triplicate whether the results were presented as mean ± standard deviation (Ecem Bayram et al. 2021) (Table S7). In addition to these processes, to make a comparison with inoculated MHA mediums, non‐inoculated MHA mediums were seeded using sterile swabs and agar well diffusion tests were repeated as described above in these media (Ecem Bayram et al. 2021).

### 2.7 Determination of Half‐Maximal Inhibitory Concentration (IC_50_ )

The broth microdilution method was used to determine the half‐maximal inhibitory concentration of royal jelly samples in a sterile 96‐well microplate. Royal jelly concentration was taken as mass per volume (mg/mL) and expressed as a percent. In 96‐well microtiter plates, serial dilutions of royal jelly ranging from 20 mg/mL to 100 mg/mL were prepared. A 18 h cultures of *Bacillus cereus* , *Staphylococcus aureus* , *Listeria monocytogenes*, *Escherichia coli* , *Shigella sonnie*, and *Salmonella enterica* was standardized to 1 × 10^5^ CFU/mL, using McFarland solution. From the standardized suspension, 100 μL was added to each well. There was a positivity control, which was broth without royal jelly. The microtiter plates were incubated at 37°C for 24 h. Inhibitory concentration of each bacterial suspension at different concentrations of royal jelly was determined based on absorbance at 600 nm using a microplate reader (BioTech type 357–914,739) and calculated using the following formula.

$$\text{Inhibition}\;\left(\%\right)=\left(\frac{\text{Absorbtion of control}-\text{Absorbition of sample}}{\text{Absorbition of control}}\right)\times 100$$

Based on inhibitory concentration, the half‐maximal inhibitory concentration (IC_50_) was determined using GraphPad Prism version 10.4.2 (San Diego, USA) based on the modified methods (İzol and Turhan 2024; Yilmaz et al. 2023). Dose–response data were obtained by plotting royal jelly concentration in percentage (mass per volume or μg/mL) (log_10_‐transformed, *X*‐axis) against the corresponding biological response (percentage inhibition or activity, *Y*‐axis). The data were fitted using a nonlinear regression model based on a three‐parameter logistic (sigmoidal) equation:

$${\mathrm{IC}}_{50}=\frac{\text{Maximum}\;\text{response}}{1+10^{\left(\log{\mathrm{IC}}_{50}-X\right)}}$$

where *Y* represents the inhibition response, *X* is the log_10_ concentration of the royal jelly, and IC_50_ is the concentration producing 50% inhibition. IC_50_ values were automatically calculated by GraphPad Prism from the fitted curves (Tables S8 and S9).

### 2.8 Determination of In Vitro Antidiabetic Evaluation of Royal Jelly by α‐Amylase and α‐Glucosidase Enzyme Inhibition Assay

#### 2.8.1 Determination of Alpha Amylase Inhibition

The α‐amylase inhibitory assay was determined according to the procedure described by Siahbalaei et al. (2021) with slight modification. Two‐fold serial dilution of royal jelly to obtain concentrations of 100, 80, 60, 40, 20 mg/mL using distilled water as the diluent. Acarbose 100 mg/mL was used as a reference standard. A 100 μL aliquot of porcine pancreatic α‐amylase solution (1.0 U/mL) was mixed with 100 μL of royal jelly sample and incubated at 37°C for 10 min. Subsequently, 100 μL of 1% (w/v) soluble starch solution prepared in 20 mM phosphate buffer (pH 6.8) was added, and the mixture was incubated at 37°C for 10 min and the reaction was terminated by adding 200 μL DNSA reagent (1% DNSA in 1.6% NaOH with 30% potassium sodium tartrate) followed by boiling in a water bath for 5 min after cooling room temperature, the mixture was diluted with 900 μL distilled water and centrifuged at 3000 × g for 10 min. The absorbance of supernatant was measured at 540 nm by using a microplate reader (BioTech type 357–914,739). A control was prepared by replacing the sample with distilled water while a reagent blank (without enzyme) was used for baseline correction. All tests were performed in triplicate and the results were presented as mean ± standard deviation. Using the following formula, percentage inhibition of alpha amylase was calculated as:

$$\begin{array}{c} \alpha -\text{amylase inhibition}\;\left(\%\right)\\ =\left(\frac{\text{Absorbtion of control}-\text{Absorbition of sample}}{\text{Absorbition of control}}\right)\times 100 \end{array}$$

The IC_50_ value was calculated using interpolation from the nonlinear regression analysis of the dose–response curve based on the modified methods (İzol and Turhan 2024; Yilmaz et al. 2023) (Table S6).

### 2.9 In Vitro Test for α‐Glucosidase Enzyme Inhibition

The method used was the α‐glucosidase inhibition test method. In vitro, α‐glucosidase inhibition assay was determined following the method of Mechchate et al. (2021) with some modification. A 100 μL aliquot of royal jelly solution was mixed with 100 μL of α‐glucosidase (1 U/mL) and pre incubated for 10 min at 37°C. The reaction was initiated by adding 100 μL of 5 mM p‐nitrophenyl α‐D‐glucosidase (PNPG) as a substrate and incubated at 37°C for 20 min, and Acarbose (100 mg/mL) was used as a reference standard. The reaction was terminated by adding 200 μL of 0.1 M Na_2_CO_3_ and the absorbance of the released p‐nitrophenyl was measured at 540 nm using a microplate reader (Type 357–914,739). Tests were performed in triplicate and the results were presented as mean ± standard deviation. The following formula was used to calculate the percentage of α‐glucosidase enzyme inhibition activities.

$$\begin{array}{c} \alpha -\text{glucosidase inhibition}\;\left(\%\right)\\ =\left(\frac{\text{Absorbition of ontrol}-\text{Absorbition of sample}}{\text{Absorbtion of control}}\right)\times 100 \end{array}$$

The IC_50_ values were calculated using non‐linear regression analysis using the mean inhibitor values and were derived from plots of percent inhibition versus log inhibitor concentration (Table S5).

### 2.10 In Vitro Bioaccessibility

The bioaccessibility of royal jelly was determined by in vitro simulated digestive system according to the procedure with some modifications described by Yesiltas et al. (2014). The methodology consists of three sequential phases, including oral, gastric, and intestinal phases as proposed by Seraglio et al. (2017) with some modifications. It was prepared 10 mg/mL of royal jelly concentration in salivary fluid for initial solubility testing. After adjusting the pH to 2.0 with gastric fluid, the sample was subjected to simulated gastric digestion at pH 2.0 in the presence of pepsin at 37°C (16 g in 100 mL 0.1 M HCl). This was followed by simulated intestinal digestion in the presence of pancreatin‐bile extract mixture (4 g porcine pancreatin and 25 g porcine bile extract in 1000 mL of 0.1 M NaHCO_3_ pH 7.5) and incubated at 37°C for 2 h while being shaken at 100 × g. When the intestinal digestion simulation is completed, the supernatant (0.22 μm syringe filter) containing the bioaccessible royal jelly was filtered and separated by centrifugation using a Centrifuge digestate at 10,000 × g for 20 min at 4°C. Finally, the soluble compounds were dried with a microwave oven for 12 h, cooled in a desiccator, and their weight was measured. All tests were performed in triplicate and the results were presented as mean ± standard deviation. Percentage of bioaccessibility was calculated by the following formula (Table S1).

$$\text{Bioaccessibility}\;\left(\%\right)=\frac{\text{Concentration in supernatant}}{\text{Total compond in original sample}}\times 100$$

### 2.11 Statistical Analysis

Multiple sample runs with triplicate measurements were used to generate the data. A one‐way analysis of variance (ANOVA) was used in R version 4.5.1 statistical software to analyze the total protein content, 10‐Hydroxy‐2‐decenoic acid, antibacterial (inhibition zone), antidiabetic (percent of inhibition of α‐amylase and α‐glucosidase), and bioaccessibility of royal jelly. The Tukey test was performed for the statistical evaluation of the data obtained. All statistical analyses of IC_50_ values were analyzed using Graph Pad Prism (version 10.4.2). Results were reported as Mean ± SD and *p* < 0.05 was considered as the level of statistical significance.

## 3 Results and Discussion

### 3.1 Total Protein and 10‐Hydroxy‐2‐Decenoic Acid (10‐HDA) Content

**FIGURE 1:**
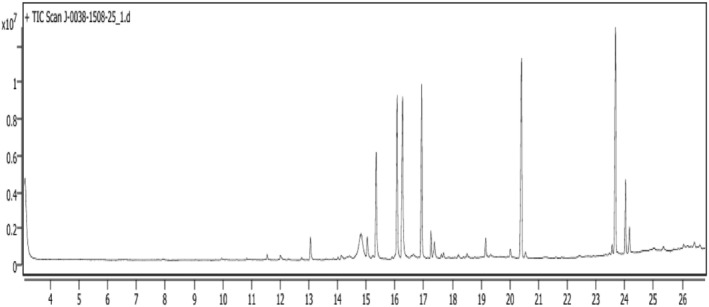
GC–MS chromatogram of 10‐hydroxy‐2‐decenoic acid in royal jelly.

For determination of total proteins in royal jelly a calibration curve was made and a logarithmic line was obtained as showed in Figure 1. El‐Guendouz et al. (2020) and Popescu et al. (2009) confirm this type of curve for Bradford method of total proteins. The calibration curve is defined by the following equation: *y* = 0.6875*x*−0.0196, *R* ^2^ = 0.9948 (Figure S1).

Gas Chromatograph—MS spectrometer results of 10‐hydroxy‐2‐decenoic acid from royal jelly were shown in Figure 1. The chromatogram obtained in analyzing of 10‐hydroxy‐2‐decenoic acid standards in a royal jelly sample was shown in Figure 1. The sample's 10‐hydroxy‐2‐decenoic acid content was calculated by comparing the corresponding peak areas to those of the pure standard solutions.

The total protein and 10‐hydroxy‐2‐decenoic acid content of royal jelly samples collected at 72 and 144 h were presented in Table 1. The harvesting time had a significant effect on the total protein and 10‐hydroxy‐2‐decenoic acid contents of royal jelly samples (*p* < 0.05).

**TABLE 1:** Total protein and 10‐Hydroxy‐2‐decenoic acid content (mean ± SD) in royal jelly collected at 72 and 144 h.

Treatments	Total protein content (%)	10‐HDA content (%)
Royal jelly (72 h)	12.99 ± 1.32^a^	3.39 ± 0.46^a^
Royal jelly (144 h)	8.36 ± 1.04^b^	1.16 ± 0.08^b^

*Note:* SD stands for standard deviation; Values in the same column with different letters are statistically different (*p* < 0.05).

The highest mean value was obtained from the samples harvested on the 72 h (12.99% ± 1.32%) while the lowest mean value was obtained in royal jelly collected on 144 h (8.36 ± 1.04). These results show similarity with Alattal et al. (2025), who are reported the protein content in royal jelly ranged from 6.73% to 13.27%. These findings were also in agreement with Emir (2020), and stated that protein content harvested in the early time and at rainy season has high protein content as compared to royal jelly collected after third day and dry season. The reduction in protein content may be due to the young larvae used more royal jelly's protein in the late harvested royal jelly as compared to the early harvested royal jelly and supported by Emir (2020). Therefore, the total protein content in royal jelly was high, collected at 72 h and lower at 144 h, and it may be for the first 3 days, all bee larvae are feed a protein rich royal jelly to fully rapid growth and cell division and to develop exclusively for queen and supported by Zheng et al. (2011).

The 10‐Hydroxy‐2‐decenoic acid (10‐HDA) content in royal jelly harvested at 72 h (3.39% ± 0.46%) was higher than that harvested at 144 h (1.16% ± 0.08%) and were significantly different (*p* < 0.05). The reduction of 10‐hydroxy‐2‐decenoic acid content over time explains the higher need of 10‐hydroxy‐2‐decenoic acid by young larvae than older larvae. Relatively similar data (0.33%–2.54%) were reported by Alattal et al. (2025). Ucak Koc et al. (2023) also reported that late harvested royal jelly's 10‐hydroxy‐2‐decenoic acid value was decreased from 3.25% to 2.24%. Royal jelly is rich in short chain (C_8_ and C_10_) hydroxy and dicarboxylic fatty acids, with 10‐hydroxy‐2‐decenoic acid as the highest amount and primary bioactive compound (Collazo et al. 2021). As a result, royal jelly harvested at 72 h had higher 10‐hydroxy‐2‐decenoic acid values than royal jelly collected at 144 h. This demonstrated that the time of harvest had an impact on the 10‐hydroxy‐2‐decenoic acid composition of royal jelly, with early harvests (72 h) having the highest quality.

### 3.2 The Antimicrobial Properties of Royal Jelly

The mean diameters of the inhibition zones for each strain by royal jelly samples collected at 72 and 144 h are shown in Table 2. This royal jelly samples collected at 72 and 144 h with mean inhibition zone diameters of *S. aureus* (11.83 mm) and (7.50 mm), *S. enterica* (1.33 mm) and (1.00 mm), *E. coli* (5.83 mm) and (1.00 mm), *B. cereus* (12.33 mm) and (7.00 mm), *L. monocytogenes* (8.00 mm), and (5.00 mm), *S. sonnie* (0.67 mm) and (1.17 mm), respectively, was effective against those strains (Table 2).

**TABLE 2:** The inhibition zone (mean ± SD) diameters (mm) for samples collected at 72 and 144 h.

Treatments	Microorganisms and inhibition zone					*S. sonnie*
	*S. aureus*	*S. enterica*	*E. coli*	*B. cereus*	*L. monocytogenes*	
Royal jelly (72 h)	11.83 ± 0.76^a^	1.33 ± 0.29^a^	5.83 ± 0.00^a^	12.33 ± 1.53^a^	8.00 ± 1.00^a^	0.67 ± 0.58^a^
Royal jelly (144 h)	7.50 ± 0.50^b^	1.00 ± 0.00^a^	1.00 ± 0.76^b^	7.00 ± 1.00^b^	5.00 ± 1.00^b^	1.17 ± 0.29^a^

*Note:* SD stands for standard deviation; Values in the same column with different letters are statistically different (*p* < 0.05).

The results of the royal jelly's antibacterial activity at 72 and 144 h were displayed in Table 2. The results of the findings showed that royal jelly samples had antibacterial effects on all Gram‐positive and Gram‐negative bacteria when the concentration was high. The antimicrobial properties of samples of royal jelly taken from the beehive at the 72 h was higher than that of samples taken on the 144 h (*p* < 0.05), while the difference between the 72 and 144 h samples was not statistically significant (*p* > 0.05) for *S. enterica and S. sonnie*. Compared to other strains of the bacteria, *S. sonnie* strains were less susceptible to royal jelly samples collected at 72 and 144 h, and the inhibition zones surrounding them were smaller and showed no significant differences (*p* > 0.05).

Royal jelly collected at 72 h exhibited the strongest antibacterial activity against *B. cereus* (12.33 mm) and *S. aureus* (11.83 mm), which contrasts with the report of Maželienė et al. (2022) that royal jelly had no antibacterial effect on *B. subtilis* and *B. cereus* spore bacteria, and the antibacterial effect of honey and honey royal jelly mixture was weak. According to Phonmat et al. (2025), the strongest inhibitory activity was *S. aureus* (11.81 ± 5.01 mm), followed by *B. cereus* F4810/72 (10.78 ± 2.71 mm), *E. coli* O157:H7 (9.38 ± 1.95), and *B. cereus* DSM4384 (6.90 ± 3.55 mm), and this is in agreement with the current study. Royal jelly contains many native and derived proteins divided into major royal jelly proteins, peptides and free amino acids demonstrating the effect of honey bee enzymes such as endopeptidases and exopeptidases on the final composition of royal jelly (Hamledari et al. 2025). Many of these active peptides have antibacterial effects. It has also been found that the components of royal jelly include many antimicrobial peptides, such as royalactin, apisimin, jeleines I, II, III, IV, apalbumin α, and 10‐hydroxy‐2‐decenoic acid, which decreased their composition when harvesting time was delayed (Hu et al. 2021; Alreshoodi and Sultanbawa 2015).

Royal jelly had the weakest antibacterial effect on *S. sonnie*, *S. enterica* , and *E. coli* and these findings correspond with previous studies (Venkateshwarlu 2022). This is due to *S. sonnie*, *S. enterica* , *and E. coli* having a high degree of intrinsic resistance to the antibacterial properties contained in royal jelly due to their complex, double‐layered cell walls, which significantly reduces the effect of the royal jelly when compared to other species (Obeidat et al. 2024).

Therefore, the antibacterial activity of royal jelly samples collected at the 72 and 144 h was found to be strongly influenced by the time of harvest, and this is in agreement with previous reports of (Mohammad et al. 2022). It seems that the antibacterial activity was related to the harvesting time of royal jelly. According to Ma et al. (2021), the harvesting time of royal jelly significantly influences its chemical composition, and it was found that royal jelly harvested at 24 h had higher levels of many bioactive compounds and lower water content compared to 48 and 72 h harvests, suggesting superior quality to inhibit microorganisms. The antibacterial potency of royal jelly is attributed to components such as 10‐hydroxy‐2‐decenoic acid, royalisin, and jelleins, which disrupt bacterial cell membranes and induce oxidative stress (Turbatmath et al. 2025). It is also crucial to notice that variations of harvesting time in royal jelly's metabolic activity affect the components and characteristics of royal jelly products. As a result, these items' antibacterial properties will vary substantially. This report is aligned with Obeidat et al. (2024), the components and characteristics of bee natural products alter from season to season due to variations in nectars, botanical sources, climate, and bee metabolic activity, and these products will have very different antibacterial qualities.

### 3.3 Inhibitory Effects of Royal Jelly on Pathogenic Bacteria

**FIGURE 2:**
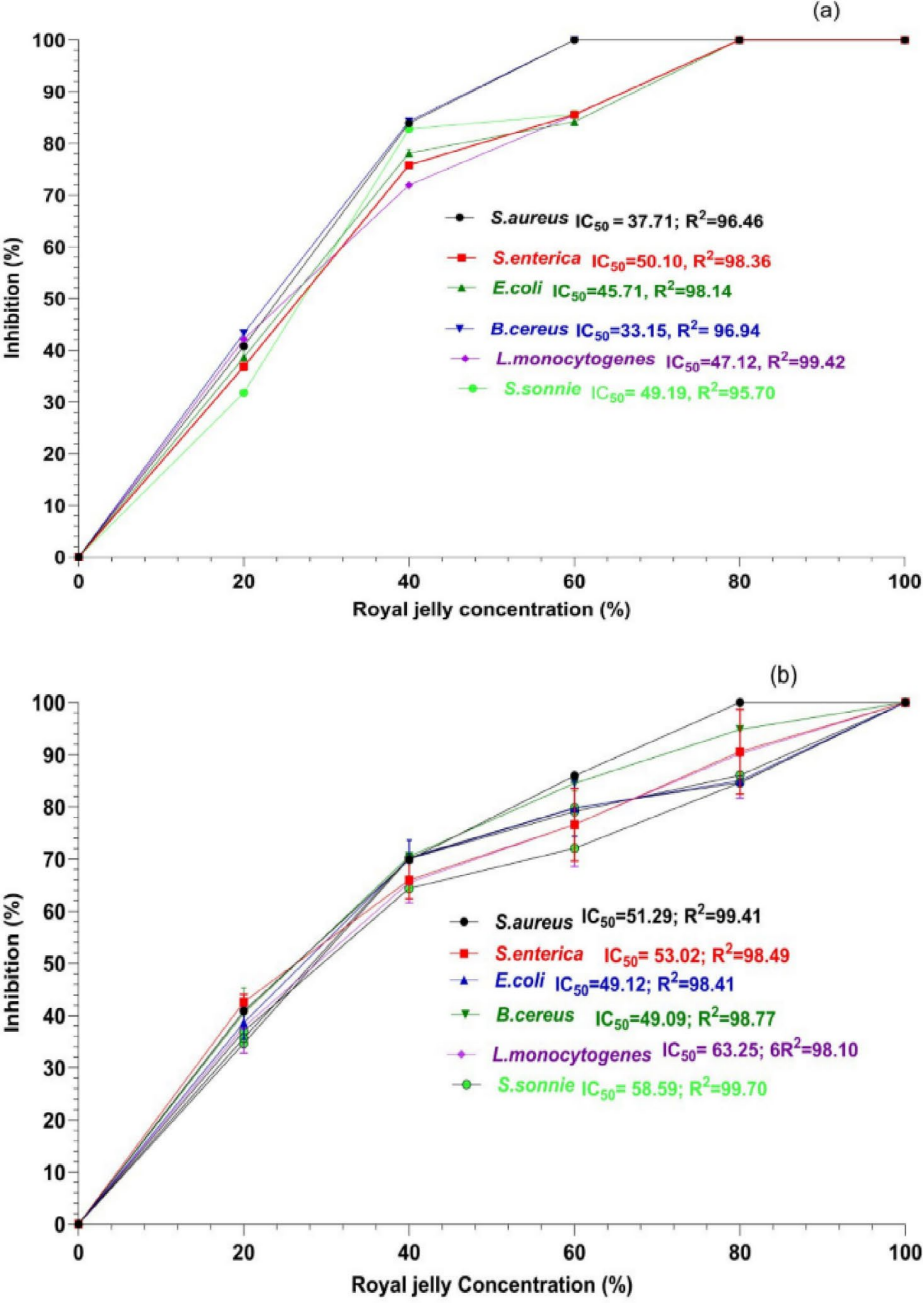
IC_50_ values of royal jelly collected at (a) 72 h and (b) 144 h on pathogenic bacteria.

Three pathogenic Gram‐positive bacteria ( *Bacillus cereus* NCCB100294, *Staphylococcus aureus* NCCB100294, *Listeria monocytogenes* NCCB100677) and three pathogenic Gram‐negative bacteria ( *Escherichia coli* NCCB72002, *Salmonella enterica* NCCB100284, and *Shigella sonnie* CCUg68726T) were used to test the antibacterial activity of royal jelly collected at 72 h. The results in Figure 2a,b showed that inhibitory effects of royal jelly were effective as the concentration was increased. At higher concentrations, the analogs showed maximum potential and inhibition of the growth of the bacteria.

The results of the half‐inhibitory concentration (IC_50_) were showed in Figure 2a. Through the comparison of the results presented in figure, it was observed that 37.71%, 50.10%, 45.71%, 33.15%, 47.12% and 49.19% in the IC_50_ values of *Staphylococcus aureus* NCCB100294, *Salmonella enterica* NCCB100284, *Escherichia coli* NCCB72002, *Bacteria cereus* NCCB100294, *Listeria monocytogenes* NCCB100677, *Shigella sonnie* CCUg68726T inhibiters, respectively. In terms of the IC_50_ value, all the analogs were good to moderate antimicrobial pathogens. The high potent of royal jelly exhibited against *B. cereus, with* an IC_50_ value of 33.15%, the while the lowest potent was observed against *S. enterica* with an IC_50_ value of 50.10%.

For *S. aureus* and *B. cereus* , the minimum inhibitory concentration was 60 mg/mL, as indicated in Figure 2a. Bacterial growth was totally inhibited at this concentration. The findings of this study align with previous research indicating that royal jelly shows a broad spectrum of antibacterial activity, particularly against Gram‐positive bacteria (Turbatmath et al. 2025). The potential of royal jelly as a natural antibacterial agent is highlighted by its capacity to inhibit the growth of both organisms, especially at similar concentrations.

Royal jelly's complex composition, which contains proteins, lipids, vitamins, and minerals in addition to bioactive substances including fatty acids (10‐hydroxy‐2‐decenoic acid), phenolic acids, and flavonoids, is thought to be responsible for its antibacterial activity during early harvesting and this is in line with the report of Hu et al. (2021). According to Bărnuţiu et al. (2011), 10‐hydroxy‐2‐decenoic acid could effectively inhibit the growth of bacteria. In the present study as shown in Figure 2a, the minimum inhibitory concentration of royal jelly against *S. sonnie, S. enterica*, *and E. coli* was found to be 80 mg/mL. These values showed that a higher concentration of royal jelly collected at 72 h is necessary to stop the growth of *S. sonnie, S. enterica*, *and E. coli*. This might result from variations in the thickness and composition of the gram‐negative bacteria's cell walls. The bioactive components of royal jelly may be unable to penetrate the thicker layer of bacteria, necessitating a higher concentration to achieve bacterial effects and in line with Ratajczak et al. (2021).

As shown in Figure 2b, IC_50_ values of royal jelly collected at 144 h royal jelly inhibit bacterial pathogens in a dose‐dependent manner at concentrations ranging from zero to 100 mg/mL. Royal jelly harvested at the 144 h inhibited pathogenic bacteria with IC_50_ values of 51.29% ( *Staphylococcus aureus* NCCB100294), 53.02% ( *Salmonella enterica* NCCB100284), 49.12% ( *Escherichia coli* NCCB72002), 49.09% ( *Bacillus cereus* NCCB100294), 63.25% ( *Listeria monocytogenes* NCCB100677), and 58.59% (*Shigella sonnie* CCUg68726T), as the results showed in Figure 2b. Among the respective IC_50_ values, high potency of royal jelly was observed for *B. cereus* (49.09%), while the lowest potency was shown in *L. monocytogenes* , with an IC_50_ value of 63.25%. This might be because the two bacteria's cell walls differ in thickness and composition. This could also explain why *L. monocytogenes* needed more royal jelly to achieve inhibitory effects. Although the precise effectiveness of royal jelly harvested at 144 h may vary depending on the species examined and their unique resistance mechanisms, the lower IC_50_ values for *B. cereus* in comparison to *L. monocytogenes* and other pathogens imply that it may be more efficient against some Gram‐positive bacteria. The results of this investigation align with previous studies showing that royal jelly had a wide range of antibacterial activities, especially against Gram‐positive bacteria. Studies have demonstrated royal jelly's inhibitory effects against *E. coli* , *Listeria monocytogenes* , and *Bacillus subtilis* , among others, Turbatmath et al. (2025) which is in agreement with the results of this study.

### 3.4 Antidiabetic Evaluation of Royal Jelly by Alpha Glucosidase and Alpha Amylase Inhibition

The in vitro α‐amylase and α‐glucosidase inhibitory activities of royal jelly collected at 72 and 144 h exhibited considerable α‐amylase and α‐glucosidase inhibitory activity (Table 3) (Table S4). The percentage inhibition of α‐amylase ranged from 75.33% to 63.57% of the highest to lowest level, while that of α‐glucosidase ranged from 75.93% to 66.56% of the highest to lowest level observed in royal jelly collected at 72 and 144 h, respectively.

**TABLE 3:** Percentage inhibition (mean ± SD) of royal jelly collected at 72 h and 144 h on α‐amylase and α‐glucosidase inhibition.

Treatments	α‐amylase inhibition	α‐glucosidase inhibition
Royal jelly 72 h	75.33 ± 0.21^a^	75.93 ± 1.05^a^
Royal jelly 144 h	63.57 ± 0.42^b^	66.56 ± 0.15^b^

*Note:* SD stands for standard deviation; Values in the same column with different letters are statistically different (*p* < 0.05).

The percentage of royal jelly inhibition collected at 72 and 144 h on the inhibition of α‐amylase and α‐glucosidase enzymes was found to be significant (*p* < 0.05) as indicated by the results in Table 3.

Royal jelly is the most potent inhibitor of α‐amylase and α‐glucosidase, which break down plant‐derived polysaccharides and are likely not harmful for the control of hyperglycemia. The results showed that the antidiabetic activity of early harvested royal jelly at 72 h possesses higher α‐amylase and α‐glucosidase inhibition as compared to royal jelly harvested at 144 h.

The potential antidiabetic effects of royal jelly were attributed to a wide range of nutrients and bioactive substances, as well as changes in chemical composition with varying harvesting times.

Zheng et al. (2011). Watadani et al. (2017) suggest that 10‐hydroxy‐2‐decenoic acid is involved in the improvement of type 2 diabetes, at least in part via activation of peroxisome proliferator‐activated receptor gamma coactivator 1 alpha (*Pgc‐1α*) expression. Daudu (2019) reported that foods like bee products with high polyphenol content have anti‐diabetic potentials. There are several mechanisms in which dietary polyphenols and bioactive compounds in royal jelly have the ability to prevent diabetes, and both preclinical and clinical data support this claim (Sun et al. 2020). Their potential inhibition of important digestive enzymes, such as α‐glucosidase and α‐amylase, slows or blocks the digestion of carbohydrates and lowers postprandial hyperglycemia, helping to control blood glucose levels (Sun et al. 2020).

Effective management of diabetes mellitus involves mild inhibition of pancreatic α‐amylase activity and substantial inhibition of intestinal α‐glucosidases, which in turn regulate postprandial plasma glucose levels and avert possible chronic vascular problems; this is aligned with Kwon et al. (2008). According to Kumavat et al. (2012) and Uddin et al. (2022), inhibitors of α‐amylase and α‐glucosidase derived from honeybee products and plants with excellent antioxidative potentials are attractive alternatives for diabetic patients. Therefore, royal jelly had effective antidiabetic properties on α‐glucosidase and α‐amylase inhibition that were collected at at 72 h as compared to the 144 h.

### 3.5 Anti‐Diabetic Evaluation of Royal Jelly on α‐Amylase and α‐Glucosidase Enzyme Inhibition (IC_50_ )

**FIGURE 3:**
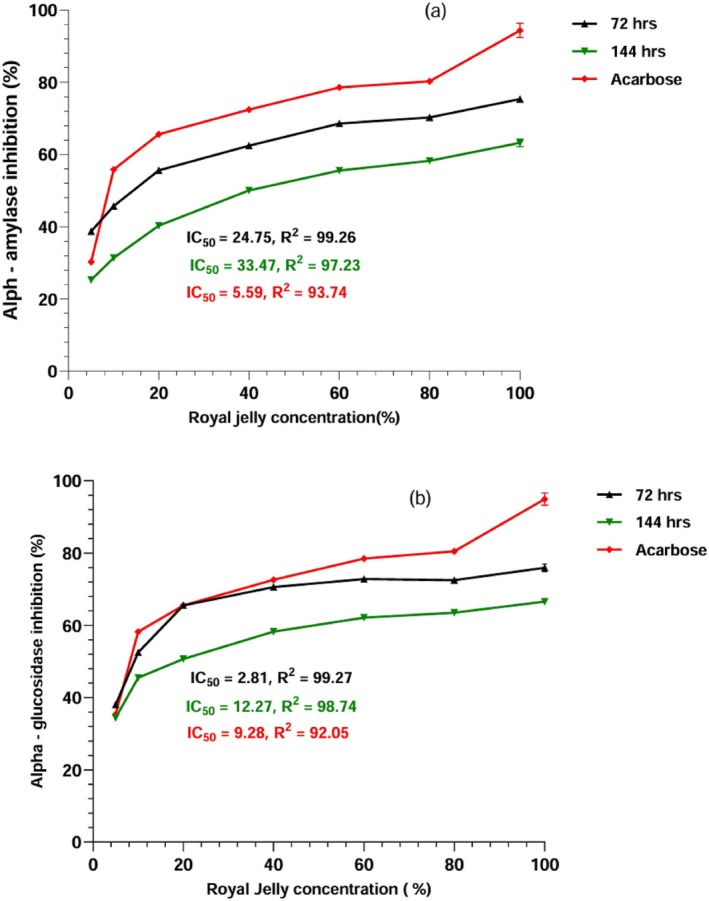
IC_50_ values of royal jelly collected at 72 h and 144 h on (a) α‐amylase and (b) α‐glucosidase enzymes inhibition (values were described as % (mg/mL)).

The results in plot showed the percentage inhibition of acarbose and royal jelly collected at 72 h and 144 h against α‐amylase. The concentration of royal jelly increased as the value of α‐amylase inhibition varied as shown in Figure 3a.

Compared to acarbose, reference compound, using IC_50_ values (resulting in 50% inhibition of enzyme activity), it was estimated to be 5.59% for acarbose and 24.70% and 33.87% for royal jelly collected at 72 h and 144 h, respectively, as shown in Figure 3a. Royal jelly collected at 144 h had IC_50_ value (33.87%) indicating the weakest effect of inhibiting activity against α‐amylase as compared to IC_50_ value (24.70%) of royal jelly harvested at 72 h. Based on the above results, royal jelly harvested at 72 h had greater inhibitory effect and was more potent on key enzymes involved in glucose metabolism, and thus the higher the potential for antidiabetic activity.

The royal jelly collected at 72 h and 144 h displayed more potent inhibitors of α‐amylase breakdown; plant‐derived polysaccharides are likely not harmful for the regulation of hyperglycemia. According to Xiao and Hogger (2015), it has been reported that foods with high polyphenol content have anti‐diabetic potentials. Polyphenols for diabetic mellitus preserve Langerhans cell islets against glucose overdose, are anti‐inflammatory, scavenge free radicals, restrict α‐amylases or α‐glucosidase activity, and reduce the formation of reactive derivatives of non‐enzymatic glucose‐protein condensation reactions (Daudu 2019).

Ghosh and Jung (2024) reported that major royal jelly proteins, phenolic compounds, peptides, fatty acids (10‐hydroxy‐2‐decenoic acid), and amino acids are bioactive compounds found in royal jelly. However, the maximum concentrations of bioactive compounds are found in early (72 h) harvested royal jelly (Emir 2020; Ma et al. 2021). Those major bioactive compounds are mainly responsible for the biological potential of the antidiabetic activities (Fallah et al. 2022). According to Fallah et al. (2022) and Gong et al. (2020), the mechanisms of action of bioactive compounds in antidiabetic activities are that amylase may interact with all amino acids, but particularly with branched‐chain amino acids (valine, leucine, and isoleucine), cyclic amino acids (proline), and aromatic amino acids (phenylalanine, tryptophan, and tyrosine). By acting as carbohydrate blockers or reversibly occupying the sugar‐binding site of amylase, low molecular weight amino acids can impede the digestion and absorption of carbohydrates in the gastrointestinal diet. By attaching to allosteric sites, high‐molecular‐weight amino acids including branched‐chain, cyclic, and aromatic amino acids can cause an enzyme's conformation to alter, decreasing the enzyme's affinity for starch and, consequently, the breakdown of polysaccharides (Fallah et al. 2022; Gong et al. 2020).

The percentage inhibition of α‐glucosidase activity depends on the concentration of royal jelly and the reference acarbose as shown in Figure 3b. For comparison, IC_50_ values of royal jelly samples were relatively close to IC_50_ of acarbose (9.28%). From the above results of the dose–response curve, it was observed that the royal jelly collected at 72 h had high potential for antidiabetic property (IC_50_ values, 2.81%) when compared to the 144 h (IC_50_ values, 12.27%) as shown in Figure 3b. The in vitro α‐glucosidase inhibition assay showed that royal jelly collected on 72 h was the most potent inhibiting α‐glucosidase activity and its IC_50_ was lower than royal jelly collected on 144 h. The results of IC_50_ values were related to IC_50_ (11.30) values of bee pollen extracts on diabetes α‐glucosidase enzymes (Daudu 2019).

A concentration‐dependent decrease in percentage inhibition was seen in the percentage inhibition of various dose (%) concentrations of royal jelly collected at the 72 and 144 h. As a result, the maximum percentage inhibition was found at the highest tested level (100%), while the lowest concentration revealed the lowest percentage, as shown in Figure 3b. The change of potential inhibitions of α‐Glucosidase enzyme by royal jelly could be variations of harvesting time of royal jelly. According to Emir (2020) and, bioactive compounds in royal jelly may be changed over time after grafting, and it could differentiate between royal jelly harvested at different times. Bioactive compounds, phenolic compounds, peptides, lipids, major royal jelly proteins, and amino acids found in royal jelly have been shown to inhibit α‐glucosidase activities, and maximum concentrations were observed in royal jelly harvested after 72 h of grafting, and this is related to other studies (Fallah et al. 2022). By slowing down the conversion of disaccharides into monosaccharides like glucose, the inhibition of these enzymes lowers the quantity of glucose absorbed into the bloodstream and prevents hyperglycemia. The competitive inhibition of the enzymes by royal jelly suggests that the inhibitory component of the bioactive compounds in royal jelly binds reversibly to the active site of the enzyme and occupies it in a mutually exclusive manner with the substrate, and is related to other studies done in honeybee pollen (Daudu 2019). Therefore, based on the above results, inhibition of α‐glucosidase activities by royal jelly at the 72 h was high potential as compared to the 144 h royal jelly.

### 3.6 Bioaccessibility of Royal Jelly

As shown in Table 4, regarding bioaccessibility of protein and 10‐Hydroxy‐2‐decenoic acid in royal jelly collected at 72 and 144 h were obtained in three phases after the simulated in vitro digestion. However, the percentage of bioaccessibility of royal jelly collected at 72 and 144 h at the oral phase was not determined.

**TABLE 4:** Bioaccessibility (mean ± SD) of royal jelly collected on the 72 and 144 h (%) at three phases after the simulated in vitro digestion.

Phases	Treatment	Total protein (%)	10‐Hydroxy‐2‐decenoic acid (%)
Oral digestion	Royal Jelly (72 h)	n.d.	n.d.
	Royal Jelly (144 h)	n.d.	n.d.
Gastric digestion	Royal Jelly (72 h)	51.49 ± 1.29^a^	58.73 ± 1.46^a^
	Royal Jelly (144 h)	46.49 ± 1.17^b^	55.02 ± 1.67^b^
Intestinal digestion	Royal Jelly (72 h)	64.36 ± 2.08^a^	64.21 ± 3.40^a^
	Royal Jelly (144 h)	55.43 ± 0.82^b^	57.21 ± 3.13^a^

*Note:* SD stands for standard deviation; Values in the same column with different letters are statistically different (*p* < 0.05); n.d stands for not digested.

Based on the bioaccessibility results in gastric digestion phase shown in Table 4, royal jelly collected at 72 h had 51.49% bioaccessible protein content and higher than that collected at 144 h (46.49%); thus, indicating that this royal jelly's protein can be used in the future for better bioaccessibility with improved biological function. According to Menezes et al. (2018) after gastrointestinal digestion, bioaccessible content of protein test results confirmed in chicken meat is 53% and is higher as compared to the percentage of bioaccessibility of protein in royal jelly in the present study.

In particular, the bioaccessibility of protein in royal jelly in the gastric stage was significantly lower compared to intestinal stage for both 72 and 144 h (*p* < 0.05). These findings were consistent with the physiological roles of digestive phases: the gastric phase (pepsin, pH 2.0) initiates protein hydrolysis producing large peptides, while the intestinal phase (trypsin, chymotrypsin, pH 6.5) further degrades these peptides into smaller peptides and free amino acids, which are more bioaccessible and related to Mureşan et al. (2018).

On the other hand, the bioaccessibility of 10‐Hydroxy‐2‐decenoic acid content in royal jelly collected at 72 and 144 h was higher in the intestinal phase than gastric phase, as shown in Table 4. This finding aligns with the physiological understanding of lipid digestion; gastric lipase initiates limited hydrolysis of fatty acids, but efficient lipid digestion requires pancreatic lipase in the intestine and is supported by Ozkan et al. (2026). However, the bioaccessible content of 10‐Hydroxy‐2‐decenoic acid in royal jelly collected at 72 h was higher than at 144 h in both gastric and intestinal stages.

The bioactive compounds in royal jelly are present when it is fresh enough or harvested early enough to have a highly available nutrient matrix, but not so mature that its components have begun to aggregate or degrade in a way that hinders its digestibility. They might create structures or complexes that are easier for intestinal and stomach enzymes to break down and digest, which would increase the amount of bioactive peptides and amino acids released; similar studies were done (Cianciosi et al. 2020).

On the other hand, according to a study performed by Yesiltas et al. (2014), samples collected from different geographical areas and seasons on propolis and pollen still have a high potential of bioaccessible bioactive compounds. The findings of Yesiltas et al. (2014) and Walle et al. (2001), indicated that even though the recovery percentages of bioaccessible bioactive compounds in propolis and pollen samples are observed to be low, the recovered amounts are still high due to their high initial contents compared to other food materials such as fruits and vegetables.

## 4 Conclusion

Royal jelly is one of the most significant functional foods that has a number of biological properties, such as antibacterial and antidiabetic ones. The variations of protein and 10‐hydroxy‐2‐decenoic acid content, antibacterial, antidiabetic activities and bioaccessibility of royal jelly samples were registered at different harvesting times. It was found that harvest time had a significant impact on the antibacterial activity of royal jelly. The α‐glucosidase and α‐amylase inhibition analysis showed that royal jelly has effective antidiabetic potential on α‐glucosidase and α‐amylase inhibition that was collected at 72 h as compared to the 144 h. Royal jelly collected at the 72 h had higher bioaccessibility of protein and 10‐hydroxy‐2‐decenoic acid content than the 144 h in gastric and intestinal stages. Thus, this study indicated that royal jelly has biological function, and more investigation will be needed to examine the biological characteristics of royal jelly in in vivo assay.

## Author Contributions

**Tadele Alemu:** conceptualization, investigation, funding acquisition, methodology, validation, visualization, writing – review and editing, formal analysis, supervision, resources. **Endale Amare:** conceptualization, writing – review and editing. **Eyuel Welelaw:** conceptualization, investigation, funding acquisition, writing – original draft, methodology, visualization, writing – review and editing, software, formal analysis, project administration, data curation, resources. **Teferi Damto:** conceptualization, writing – review and editing, resources, supervision. **Mesfin Getachew Tadesse:** conceptualization, writing – review and editing. **Admasu Addi:** conceptualization, writing – review and editing, supervision, resources. **Feriehiwote Weldeyohanis Gebremariam:** conceptualization. **Ibrahim Ahmed Sherif:** conceptualization. **Abera Belay:** conceptualization, investigation, funding acquisition, methodology, validation, visualization, software, formal analysis, project administration, data curation, supervision, resources, writing – review and editing. **Henock Woldemichael Woldemariam:** conceptualization, funding acquisition, writing – review and editing, project administration, supervision.

## Funding

The authors have nothing to report.

## Conflicts of Interest

The authors declare no conflicts of interest.

## Supporting information


**Figure S1:** Calibration curve for Bradford Method.
**Table S1:** Raw data for in vitro Bioaccessibility (%) in three phases.
**Table S2:** Raw data of 10‐hydroxy‐2decenoic acid (%).
**Table S3:** Raw data for total protein (%).
**Table S4:** Percent of α—Glucosidase and α ‐Amylase inhibition.
**Table S5:** Raw data for IC_50_ (%) (α—Glucosidase inhibition).
**Table S6:** Raw data for IC_50_ (%) (α—Amylase inhibition).
**Table S7:** Raw data for determination of in vitro antibacterial properties (inhibitory zones).
**Table S8:** Raw data for IC_50 of_ royal jelly collected at 72 h.
**Table S9:** Raw data for IC_50 of_ royal jelly collected at 144 h.

## Data Availability

The authors confirm that the data supporting the findings of this study are available within the article and its Supporting Information.
