# Supplementary material for: Total Protein and 10‐Hydroxy‐2‐Decenoic Acid Content, In Vitro Bioaccessibility, Antibacterial, and Antidiabetic Properties of Royal Jelly Collected at Different Harvesting Times
Source: Food Sci Nutr. 2026 Jul 29;14(8):e72127. doi: 10.1002/fsn3.72127 (PMC13416401; doi:10.1002/fsn3.72127)
Supplement: Supplementary file 1 — Figure S1: Calibration curve for Bradford Method. Table S1: Raw data for in vitro Bioaccessibility (%) in three phases. Table S2: Raw data of 10‐hydroxy‐2decenoic acid (%). Table S3: Raw data for total protein (%). Table S4: Percent of α—Glucosidase and α ‐Amylase inhibition. Table S5: Raw data for IC50 (%) (α—Glucosidase inhibition). Table S6: Raw data for IC50 (%) (α—Amylase inhibition). Table S7: Raw data for determination of in vitro antibacterial properties (inhibitory zones). Table S8: Raw data for IC50 of royal jelly collected at 72 h. Table S9: Raw data for IC50 of royal jelly collected at 144 h. [file FSN3-14-e72127-s001.docx]

**Figure S1:** Calibration curve for Bradford Method

| Phases | Treatments | Total protein ((%) | 10-hydroxy - 2 decenoic acid (%) | |
| --- | --- | --- | --- | --- |
| Oral digestion | Royal Jelly (72 hrs) | n.d. | n.d. |  |
|  | Royal Jelly (72 hrs) | n.d. | n.d. |  |
|  | Royal Jelly (72 hrs) | n.d. | n.d. |  |
|  | Royal Jelly (144 hrs) | n.d. | n.d. |  |
|  | Royal Jelly (144 hrs) | n.d. | n.d. |  |
|  | Royal Jelly (144 hrs) | n.d. | n.d. |  |
| Gastric digestion | Royal Jelly (72 hrs) | 51.83 | 68.11 |  |
|  | Royal Jelly (72 hrs) | 50.04 | 61.85 |  |
|  | Royal Jelly (72 hrs) | 52.59 | 62.66 |  |
|  | Royal Jelly (144 hrs) | 47.63 | 59.63 |  |
|  | Royal Jelly (144 hrs) | 46.54 | 53.60 |  |
|  | Royal Jelly (144 hrs) | 45.29 | 58.41 |  |
| Intestinal digestion | Royal Jelly (72 hrs) | 61.95 | 60.13 |  |
|  | Royal Jelly (72 hrs) | 65.43 | 57.23 |  |
|  | Royal Jelly (72 hrs) | 65.69 | 58.82 |  |
|  | Royal Jelly (144 hrs) | 56.14 | 55.96 |  |
|  | Royal Jelly (144 hrs) | 55.63 | 56.00 |  |
|  | Royal Jelly (144 hrs) | 54.52 | 53.10 |  |

**Table S1: Raw data for in vitro Bioaccessibility (%) in three phases**

**Table S2: Raw data of 10-hydroxy-2decenoic acid (%)**

| Treatments | 10-hydroxy-2decenoic acid (%) |
| --- | --- |
| Royal Jelly (72 hrs) | 3.01 |
| Royal Jelly (72 hrs) | 3.25 |
| Royal Jelly (72 hrs) | 3.91 |
| Royal Jelly (144 hrs) | 1.09 |
| Royal Jelly (144 hrs) | 1.25 |
| Royal Jelly (144 hrs) | 1.13 |

| **Table S3: Raw data for total protein (%)** | |
| --- | --- |
| Treatments | Protein (%) |
| Royal Jelly (72 hrs) | 13.14 |
| Royal Jelly (72 hrs) | 14.23 |
| Royal Jelly (72 hrs) | 11.6 |
| Royal Jelly (144 hrs) | 8.65 |
| Royal Jelly (144 hrs) | 9.24 |
| Royal Jelly (144 hrs) | 7.19 |

**Table S4: Percent of α - Glucosidase and α -Amylase inhibition**

| Treatments | a- Glucosidase inhibition (%) | a-Amylase inhibition (%) |
| --- | --- | --- |
| Royal Jelly (72 hrs) | 74.8 | 75.4 |
| Royal Jelly (72 hrs) | 76.1 | 75.5 |
| Royal Jelly (72 hrs) | 76.9 | 75.1 |
| Royal Jelly (144 hrs) | 66.6 | 63.7 |
| Royal Jelly (144 hrs) | 66.7 | 63.9 |
| Royal Jelly (144 hrs) | 66.4 | 63.1 |
| Acarbose | 95.1 | 94.2 |
| Acarbose | 93.2 | 96.4 |
| Acarbose | 96.6 | 92.5 |

**Table S5: Raw data for IC_50_ (%) (α - Glucosidase inhibition)**

| IC_50_ (%) a- Glucosidase inhibition | | | | | | | |  | |  | |  | |
| --- | --- | --- | --- | --- | --- | --- | --- | --- | --- | --- | --- | --- | --- |
| Sample  Concentration (%) | Royal Jelly  (72 hrs) | | | Royal Jelly  (144 hrs) | | | Acarbose | | | | | |  |
| 5 | 38.2 | 38.1 | 37.8 | 34.9 | 34.2 | 34.1 | 29.7 | | 30.4 | | 30.6 | |  |
| 10 | 52.4 | 53.1 | 51.9 | 45.8 | 45.6 | 45.1 | 55.7 | | 55.9 | | 55.8 | |  |
| 20 | 65.2 | 65.7 | 65.6 | 50.2 | 50.9 | 50.9 | 65.9 | | 65.7 | | 65.3 | |  |
| 40 | 70.7 | 70.2 | 70.9 | 58.3 | 58.4 | 58.13 | 72.1 | | 72.8 | | 72.5 | |  |
| 60 | 72.3 | 73.8 | 72.3 | 62.1 | 62.01 | 62.3 | 78.8 | | 78.2 | | 78.7 | |  |
| 80 | 72.6 | 72.1 | 72.8 | 63.3 | 63.7 | 63.5 | 80.6 | | 80.3 | | 80 | |  |
| 100 | 74.8 | 76.1 | 76.9 | 66.6 | 66.7 | 66.4 | 94.2 | | 96.4 | | 92.5 | |  |

**Table S6: Raw data for IC_50_ (%) (α - Amylase inhibition)**

|  | IC_50_ (%) a- Amylase inhibition | | | | | | |  |  |  |
| --- | --- | --- | --- | --- | --- | --- | --- | --- | --- | --- |
| Sample  Concentration (%) | | Royal Jelly (72 hrs) | | | Royal Jelly (144 hrs | | | Acarbose | | |
|  | 5 | 38.5 | 39.8 | 37.9 | 28.4 | 26.9 | 29.2 | 29.7 | 30.4 | 30.6 |
|  | 10 | 42.7 | 43.9 | 51.8 | 41.3 | 39.7 | 42.1 | 55.7 | 55.9 | 55.8 |
|  | 20 | 55.9 | 55.7 | 55.3 | 50.2 | 50.4 | 50.4 | 65.9 | 65.7 | 65.3 |
|  | 40 | 62.1 | 62.8 | 62.5 | 54.2 | 54.7 | 54.9 | 72.1 | 72.8 | 72.5 |
|  | 60 | 68.8 | 68.2 | 68.7 | 55.8 | 55 | 55.8 | 78.8 | 78.2 | 78.7 |
|  | 80 | 70.6 | 70.3 | 70 | 58.3 | 58.2 | 58.3 | 80.6 | 80.3 | 80 |
|  | 100 | 75.4 | 75.5 | 75.1 | 63.7 | 63.9 | 63.1 | 94.2 | 96.4 | 92.5 |

**Table S7: Raw data for determination of in vitro antibacterial properties (inhibitory zones)**

| Treatments | Pathogens | | | | | |
| --- | --- | --- | --- | --- | --- | --- |
|  | *S.aureus* | *S.enerica* | *E.coli* | *B.cereus* | *L.monocytogenes* | *S.sonnie* |
| Royal Jelly (72 hrs) | 12 | 1.3 | 6 | 12 | 8 | 0.65 |
| Royal Jelly (72 hrs) | 12.5 | 1.3 | 5 | 14 | 9 | 1 |
| Royal Jelly (72 hrs) | 11 | 1.4 | 6.5 | 11 | 7 | 0.37 |
| Royal Jelly (144 hrs) | 7.5 | 1 | 1 | 7 | 4 | 1.25 |
| Royal Jelly (144 hrs) | 8 | 1 | 1 | 6 | 5 | 1.1 |
| Royal Jelly (144 hrs) | 7 | 1 | 1 | 8 | 6 | 1.15 |

**Table S8: Raw data for IC_50_ of royal jelly collected at 72 hrs**

| Royal jelly concentration  (mg/mL) | *S.aureus* | | | *S.enerica* | | | *E.coli* | | | *B.cereus* | | | *L.monocytogenes* | | | *S.sonnie* | | |
| --- | --- | --- | --- | --- | --- | --- | --- | --- | --- | --- | --- | --- | --- | --- | --- | --- | --- | --- |
| 100 | 100.00 | 100.00 | 100.00 | 100.00 | 100.00 | 100.00 | 100.00 | 100.00 | 100.00 | 100.00 | 100.00 | 100.00 | 100.00 | 100.00 | 100.00 | 100.00 | 100.00 | 100.00 |
| 80 | 100.00 | 100.00 | 100.00 | 100.00 | 100.00 | 100.00 | 100.00 | 100.00 | 100.00 | 100.00 | 100.00 | 100.00 | 100.00 | 100.00 | 100.00 | 100.00 | 100.00 | 100.00 |
| 60 | 100.00 | 100.00 | 100.00 | 85.88 | 85.36 | 85.46 | 84.68 | 83.29 | 84.59 | 100.00 | 100.00 | 100.00 | 85.72 | 84.96 | 85.75 | 86.07 | 86.07 | 84.68 |
| 40 | 83.78 | 83.89 | 84.06 | 75.85 | 75.67 | 75.95 | 77.72 | 77.72 | 78.99 | 84.34 | 84.50 | 84.01 | 71.73 | 72.01 | 72.07 | 82.59 | 82.87 | 83.01 |
| 20 | 40.98 | 40.48 | 40.84 | 36.44 | 36.35 | 37.77 | 38.72 | 35.93 | 41.18 | 42.66 | 43.85 | 43.45 | 42.33 | 41.50 | 42.60 | 31.89 | 31.48 | 32.03 |
| 0 | 0 | 0 | 0 | 0 | 0 | 0 | 0 | 0 | 0 | 0 | 0 | 0 | 0 | 0 | 0 | 0 | 0 | 0 |

**Table S9: Raw data for IC_50_ of royal jelly collected at 144 hrs**

| Royal jelly  concentration  (mg/mL) | *S.aureus* | | | | *S.enerica* | | | | *E.coli* | | | | *B.cereus* | | | | *L.monocytogenes* | | | | *S.sonnie* | | | |
| --- | --- | --- | --- | --- | --- | --- | --- | --- | --- | --- | --- | --- | --- | --- | --- | --- | --- | --- | --- | --- | --- | --- | --- | --- |
| 100 | 100.00 | 100.00 | 100.00 | 100.00 | | 100.00 | 100.00 | 100.00 | | 100.00 | 100.00 | 100.00 | | 100.00 | 100.00 | 100.00 | | 100.00 | 100.00 | 100.00 | | 100.00 | 100.00 |  |
| 80 | 100.00 | 100.00 | 100.00 | 100.00 | | 85.88 | 85.86 | 84.44 | | 86.07 | 84.68 | 84.59 | | 100.00 | 100.00 | 100.00 | | 84.53 | 86.07 | 84.64 | | 86.07 | 84.68 |  |
| 60 | 86.01 | 86.01 | 85.99 | 84.62 | | 71.89 | 73.27 | 73.55 | | 82.87 | 82.73 | 82.91 | | 84.62 | 86.03 | 85.97 | | 72.01 | 72.01 | 72.07 | | 79.11 | 79.81 |  |
| 40 | 69.93 | 69.79 | 69.89 | 70.07 | | 63.28 | 64.64 | 66.05 | | 72.14 | 72.14 | 70.59 | | 70.35 | 70.53 | 69.99 | | 63.43 | 63.09 | 64.39 | | 70.06 | 70.33 |  |
| 20 | 41.12 | 40.98 | 40.48 | 40.84 | | 43.50 | 43.42 | 42.01 | | 38.02 | 35.93 | 34.87 | | 42.66 | 43.85 | 43.45 | | 34.32 | 35.65 | 37.01 | | 34.68 | 35.65 |  |
| 0 | 0 | 0 | 0 | 0 | | 0 | 0 | 0 | | 0 | 0 | 0 | | 0 | 0 | 0 | | 0 | 0 | 0 | | 0 | 0 |  |
